# Effectiveness of internet-delivered cognitive behavioural therapy for anxiety and obsessive-compulsive disorders within routine clinical care in rural Sweden

**DOI:** 10.1016/j.invent.2024.100738

**Published:** 2024-04-06

**Authors:** Sarah Vigerland, Sandra Fredlander, Kristina Aspvall, Maral Jolstedt, Fabian Lenhard, David Mataix-Cols, Brjánn Ljótsson, Eva Serlachius

**Affiliations:** a Centre for Psychiatry Research, Department of Clinical Neuroscience, Karolinska Institutet, Norra stationsgatan 69, 113 64 Stockholm, Sweden; b Stockholm, CAP Research Centre, Stockholm Health Care Services, Region Stockholm, Gävlegatan 22, 113 30 Stockholm, Sweden; c Barn-och ungdomspsykiatrins mottagning, Region Jämtland Härjedalen, Östersunds sjukhus, 831 83 Östersund, Sweden; d Division of Psychology, Department of Clinical Neuroscience, Karolinska Institutet, Nobels väg 9, 171 65 Stockholm, Sweden; e Department of Clinical Sciences, Faculty of Medicine, Section of Child and Adolescent Psychiatry, Lund University, Baravägen 1, Forskningsenheten, 221 85 Lund, Sweden

**Keywords:** Anxiety disorders, Obsessive-compulsive disorder, Cognitive behaviour therapy, Internet-delivered treatment, Children, Rural health services

## Abstract

Few studies have evaluated the implementation of ICBT in regular child and adolescent mental health services (CAMHS). This study aimed to explore the acceptability, feasibility, and effectiveness of ICBT for children and adolescents with anxiety disorders and obsessive-compulsive disorder (OCD) within a rural CAMHS. The study also explored outcome predictors and long-term outcomes.

Eighty-three participants were consecutively recruited from a non-specialized CAMHS in Region Jämtland Härjedalen in northern Sweden. Therapist-guided ICBT was offered during 12 weeks to children aged 8–17 with an anxiety disorder or OCD. Acceptability and feasibility measures included treatment adherence, treatment satisfaction, and adverse events. The primary outcome measure was the Clinical Global Impression–Severity. Secondary measures of effectiveness included clinician-, self-, and parent-ratings of symptom severity and functional impairment. Assessments were completed at baseline, post-treatment, and three-month follow-up (primary endpoint). A two-year follow up was conducted using medical records. Potential predictors included both patient characteristics and treatment variables.

Results indicated that ICBT was both acceptable and feasible according to study measures. Statistically significant improvements were found from baseline to the three-month follow-up on clinician rated severity (*B* [SE] = −0.92 [0.09]; *p* < .001), as well as on all secondary measures. Forty-three percent of participants no longer fulfilled criteria for their principal disorder at the three-month follow-up. No serious adverse events were reported. Clinical improvement was highest among children with higher functioning at baseline (*B* [SE] = −0.05 [0.02]; *p* < .05). Forty-six percent of participants had been in contact with CAMHS during the two-year follow-up period, mainly for reasons other than their initial diagnosis. Findings suggest that ICBT could be an acceptable and feasible treatment option for young people with anxiety disorders and OCD in rural non-specialized CAMHS settings. Further studies are needed to confirm treatment effectiveness in this setting. Trial registration: NCT02926365.

## Highlights

• ICBT was both acceptable and feasible in regular non-specialized CAMHS.
• Statistically significant improvements were found on all measures of effectiveness.
• No serious adverse events were reported.
• Treatment improvement was associated with higher functioning at baseline.

## 1 Introduction

Anxiety disorders and obsessive-compulsive disorder (OCD) are common mental health disorders in children and adolescents and are associated with functional impairment and increased risk of adverse outcomes in adulthood (e.g., Copeland et al., 2014; Polanczyk et al., 2015; Pérez-Vigil et al., 2019). Although cognitive behavioural therapy (CBT) is effective for these disorders and recommended as the first-line treatment in clinical guidelines (James et al., 2020; Reynolds et al., 2012; Uhre et al., 2020), accessibility to these evidence-based treatments is low, partly because of limited access to CBT therapists (Shafran et al., 2009; Cavanagh, 2014). For example, in England, Reardon et al. (2020) found that <3 % of families seeking help for anxiety disorders had received CBT.

Internet-delivered CBT (ICBT) has been suggested as a way of increasing treatment availability (Hedman et al., 2012), and has been evaluated for anxiety and OCD in children and adolescents with promising results (Grist et al., 2019; Vigerland et al., 2016a, Vigerland et al., 2016b). In three studies reporting on the effectiveness of ICBT for anxiety disorders in non-specialist child and adolescent mental health services (CAMHS; Silfvernagel et al., 2015; Waite et al., 2019; Moor et al., 2019), a large proportion of children improved on measures of anxiety, although the difference in improvement between the ICBT and waitlist group in Waite et al. (2019) was not statistically significant. Participants who completed more sessions and had elevated anxiety at baseline seemed to improve more from treatment (Waite et al., 2019), which highlights the need of further research to establish how to optimize ICBT outcomes.

Our research team has previously evaluated ICBT programs for children and adolescents with anxiety disorders (“BIP Anxiety”) and OCD (“BIP OCD”) in a series of open and randomized controlled trials (Aspvall et al., 2018; Aspvall et al., 2020; Aspvall et al., 2021a, Aspvall et al., 2021b; Jolstedt et al., 2018a; Jolstedt et al., 2018b; Lenhard et al., 2014; Lenhard et al., 2017; Vigerland et al., 2013; Vigerland et al., 2016a, Vigerland et al., 2016b; Wickberg et al., 2022). Jolstedt et al. (2018a) was, unlike our other previous studies, conducted at a non-specialist CAMHS. While Jolstedt et al. (2018a) showed promising results, the sample size was very small.

The aim of the present study was to conduct an open trial with a naturalistic follow-up to evaluate the acceptability, feasibility and potential effectiveness of ICBT for children and adolescents with anxiety disorders and/or OCD within a non-specialized CAMHS, and to explore outcome predictors and long-term outcomes. With very few exclusion criteria, we aimed to mimic how ICBT might be used if it were broadly disseminated within a non-specialized CAMHS in a rural region. Specifically, we aimed to answer the following research questions:

1) Is ICBT an acceptable and feasible treatment for children and adolescents with anxiety disorders and OCD in a non-specialized CAMHS setting?
2) Is ICBT an effective treatment in non-specialized CAMHS?
  a) Is there a need for further treatment after completing ICBT?
  b) What are the long-term effects of ICBT?
  c) For which patient groups is ICBT most effective?

On the basis of the existing literature, we hypothesized that ICBT would be acceptable, feasible and potentially effective in this setting. No hypotheses were formulated regarding need for further treatment, long-term effects and outcome predictors.

## 2 Methods

### 2.1 Design

This was an open trial with a naturalistic follow-up at the CAMHS in Region Jämtland Härjedalen, Sweden, with consecutive inclusion during a predefined period from October 1st, 2016, to September 30th, 2018. The region is one of the most rural in Sweden with a population density of 2.6 inhabitants per square kilometre (compared to the national average of 24.2; OECD, 2017). The study was approved by the Ethical review board in Stockholm (DNR 2016/1355-31/5; 2016/2142-32). Parents provided written consent and children and adolescents provided verbal consent before being included in the study. Families provided separate consent for a two-year follow-up using medical records. The study was registered at ClinicalTrials.gov (NCT02926365).

### 2.2 Participants

To be eligible for the trial, participants had to a) be 8–17 years of age, b) fulfil diagnostic criteria for an anxiety disorder (generalized anxiety disorder, separation anxiety disorder, social anxiety disorder, panic disorder, agoraphobia or specific phobia) or OCD, c) have access to internet at home, d) be able to read and write in Swedish. Finally, e) one parent or caregiver had to be able and willing to participate in the study. Exclusion criteria were held to a minimum and large emphasis was placed on whether the intervention was deemed appropriate by the assessing clinician and the team of study clinicians (including the first author) given the patient's current situation. Anxiety or OCD did not have to be the patient's primary disorder. Acute symptoms requiring immediate care (e.g., severe depression or high risk of suicide) and/or psychosocial problems (e.g., substance abuse or maltreatment) led to exclusion.

### 2.3 Procedure

Children and adolescents were recruited consecutively from CAMHS in Region Jämtland Härjedalen. Patients who contacted the CAMHS with a suspected anxiety disorder or OCD, were informed about the study by the intake team and subsequently referred to the study coordinator if interested in participation. Existing patients at the clinic could also be referred. An initial telephone screening was conducted with a caregiver, where questions about the study were answered, and inclusion and exclusion criteria were briefly checked. A baseline assessment, where psychiatric diagnoses were assessed using the semi structured interview Mini International Neuropsychiatric Interview for Children and Adolescents (MINI-KID; Sheehan et al., 2010), was conducted at the clinic with eligible participants. If OCD was the principal disorder to be targeted in treatment the assessment also included the Children's Yale-Brown Obsessive-Compulsive Scale (CY-BOCS; Scahill et al., 1997). Included participants were given access to either the BIP Anxiety or BIP OCD after providing consent and completing self- and parent-rated questionnaires.

Assessments were conducted at the clinic before treatment (baseline), after 12 weeks when the treatment period was completed (post-treatment) and 12 weeks after post-treatment (three-month follow-up; primary endpoint). Self- and parent-rated measures were administered online at the same assessment points. The families had access to the ICBT material without therapist contact during the follow-up period. If participants had consented to long-term follow-up, the first author extracted the information from the participants' CAMHS medical record two years after treatment.

Six licensed mental health professionals (five psychologists and one counsellor) working as clinicians at the CAMHS conducted the assessments and provided therapist-support. All of them had at least basic training in CBT and they had on average 6.8 years of experience of clinical work (SD 8.0; range 0.5–18). They received a 2-day training in assessment and treatment content prior to study start, had weekly or biweekly supervision with a licensed psychologist with five years' experience of ICBT and treatment of the disorders (first author) and could also receive supervision on demand.

### 2.4 Intervention

The treatments, BIP Anxiety and BIP OCD, have been described in detail previously (Jolstedt et al., 2018b; Aspvall et al., 2021a, Aspvall et al., 2021b). For the purpose of this study, BIP OCD was shortened from 14 to its original 12 modules (Lenhard et al., 2017; Aspvall et al., 2018), and an adolescent version of the BIP Anxiety program for children was created with age-appropriate examples and parent content (inspired by the parent modules in Wahlund et al., 2020). Both programs consist of 12 modules comprising informative texts, online videos, interactive exercises, and worksheets. See Supplementary Table S1 for detailed treatment content.

The therapists logged in three times a week to answer questions, and comment on worksheets and homework assignments. Therapist support was provided through the digital platform, but therapists could call participants or propose a meeting at the clinic if the therapists deemed it necessary from a clinical perspective (e.g., assess suicidal ideation). Therapists also reached out to participants through text messages or phone calls if they were not engaging with the program.

### 2.5 Measures

#### 2.5.1 Acceptability

Acceptability was measured by the proportion of patients who accepted the offer of ICBT and the proportion of participants who dropped out of treatment. Reasons for dropping out of treatment were logged by the therapist.

Treatment adherence was measured at post-treatment by number of completed modules and the clinician rated questionnaire Internet Intervention Patient Adherence Scale (iiPAS; Lenhard et al., 2019). Treatment credibility was measured three weeks after starting the program, using the Treatment credibility and expectancy – child and parent version (C-scale; Borkovec and Nau, 1972). See Supplement 1 for more detailed information.

Treatment satisfaction was measured at post-treatment by asking participants and parents if they learned how to better deal with their/their child's anxiety, whether the material was age appropriate, what they thought about the therapist contact, and if they would have preferred face-to-face treatment. An additional question, asking participants to give the treatment an overall grade, was answered on a four-point scale from 0 =“Bad” to 3 =“Great”. These questions were based on previous experiences and chosen to reflect program satisfaction.

#### 2.5.2 Feasibility

Several measures of feasibility were used in the trial. Clinician time per participant was automatically registered in the treatment platform. Number of telephone calls, visits to the clinic and other deviations from ordinary treatment procedures was logged by therapists in an electronic document on a weekly basis.

Participants and their parents were asked at post-treatment to describe any adverse events (from the participants' perspective) during treatment, as well as the event's past and current impact on the participant's wellbeing. Severity of events was evaluated by the first author.

#### 2.5.3 Effectiveness

##### 2.5.3.1 Primary outcome measure

The primary outcome measure was the Clinical Global Impression–Severity (CGI—S; Guy, 1976). The CGI-S is rated by the clinician on a seven-point scale from 1 = “Normal, not at all ill” to 7 = “Among the most extremely ill patients”. The scale has shown stability over time as well as sensitivity for detecting change for psychiatric disorders (Berk et al., 2008; Zaider et al., 2003; Leon et al., 1993). In this study, a CGI-S rating was assigned to the disorder that was targeted in treatment. If a principal disorder could not be defined, i.e., more than one disorder was rated with the same level of severity, this was registered as mixed anxiety and the disorder with the highest score at post-treatment was used as the principal disorder in the analyses. Remission was defined as having a CGI-S score ≤ 3 (corresponding to borderline or not at all ill) on the principal disorder.

##### 2.5.3.2 Secondary outcome measures

Clinician rated secondary outcome measures were Clinical Global Impression–Improvement (CGI—I; Guy, 1976) and the Children's Global Assessment Scale (CGAS; Shaffer, 1983; Lundh et al., 2010). Children and parents completed questionnaires on symptoms and functioning online using the anxiety subscale of the Revised Children's Anxiety and Depression Scale–Child and parent version (RCADS-C/P; Chorpita et al., 2005) and the Work and Social Adjustment Scale–youth and parent version (WSAS-Y/P; Jassi et al., 2020). When OCD was the disorder targeted in treatment, the CY-BOCS (Scahill et al., 1997) was administered to assess OCD symptom severity. At the three-month follow-up assessment, the clinician recorded whether the patient needed further treatment or could be discharged from the clinic. Detailed information on secondary outcome measures can be found in Supplement 1.

##### 2.5.3.3 Long-term effects

Long-term effects were evaluated two years after treatment completion and were defined as need of, and reason for, further contact with the CAMHS clinic. This information, together with number of visits at the clinic, was extracted manually by author SV from the participants' medical records for the participants who had given consent for this part of the study. No assessments or self-report measures were conducted as part of the long-term follow-up.

### 2.6 Data analysis

As this was a naturalistic evaluation, no power calculation was conducted a priori. All statistical analyses were done using SPSS version 28. Prior to analyses, variables were screened for missing data, outliers and normality. No cases were excluded from the analyses. A series of linear mixed model analyses (LMM; Hesser, 2015) were used to detect statistical change from baseline to three-month follow-up (primary endpoint). Baseline (coded = 0), post-treatment (coded = 1) and follow-up (coded = 2) were entered into the models. Random effects for intercept and time were added stepwise until the best fitting model was found. Using all available data, mixed-models analysis is capable of handling missing data as long as it is missing at random (Gueorguieva and Krystal, 2004). No data imputation was conducted. Supplementary completer analyses were performed for all outcomes of clinical effectiveness using paired samples *t*-tests.

Effect sizes (Cohen's *d*) for the changes between baseline and three-month follow-up were determined from the coefficient for the slope divided by the baseline standard deviation (Feingold, 2009). Effect sizes were categorized as small if *d* > 0.2, medium if *d* > 0.5 and large if *d* > 0.8. All effect sizes are presented so that positive scores indicate improvement. Rates for further need of treatment, improvement, and remission were calculated using observed data.

To assess predictors of improvement at primary endpoint, CGI-I was used as the dependent variable (data from completers only). Pearson's correlations and *t*-tests were used to explore associations with continuous and categorical variables, respectively. Scatterplots were used to visually inspect if non-linear associations existed. Significant variables were then entered simultaneously in a multiple regression. Potential predictors comprised both baseline variables (age, gender, duration of disorder, baseline scores of clinician-, self- and parent-reported outcomes) as well as in treatment variables (completed chapters, therapist time, and clinician rated adherence).

## 3 Results

### 3.1 Baseline characteristics and study flow

Of 107 patients informed of the study at first contact with the clinic, 13 (12 %) declined to receive further information. Eighty-three children and adolescents were included in the study. Baseline characteristics are presented in Table 1. Most participants were adolescents (68 %) and had a principal anxiety disorder (78 %).

**Table 1:** Socio-demographic and clinical data.

	Total sample (*N* = 83)
Females, *n* (%)	64 (77.1)
Age, *M (SD)*	13.43 (2.51)
Parental educational level, *N* (%)	
Up to 12 years of education	22 (26.5)
University, <3 years	14 (16.9)
University, 3 years or more	44 (53.0)
Distance from home to clinic, *M (SD)*; median	50.34 (62.7); 22.0
Principal disorder, N (%)	
Mixed anxiety	19 (22.9)
Obsessive-compulsive disorder	18 (21.7)
Social anxiety disorder	18 (21.7)
Specific phobia	10 (12.0)
Panic disorder	8 (9.6)
Generalized anxiety disorder	5 (6.0)
Separation anxiety disorder	4 (4,8)
Agoraphobia	1 (1.2)
Depressive symptoms^a^, *M (SD)*	11.94 (7.0)
Duration of disorder in years, *M (SD)*	3.45 (2.79)
On psychotropic medication, *N* (%)	24 (31.2)
ADHD, *N* (%)	
Suspected	19 (22.9)
Diagnosed	6 (7.2)
ASD, *N* (%)	
Suspected	9 (10.8)
Diagnosed	6 (7.2)

ADHD = Attention deficit and hyperactivity disorder. ASD = Autism spectrum disorder. NB. Mixed anxiety = two anxiety disorders with equal severity.

a Depression subscale of RCADS-C.

**Fig. 1:**
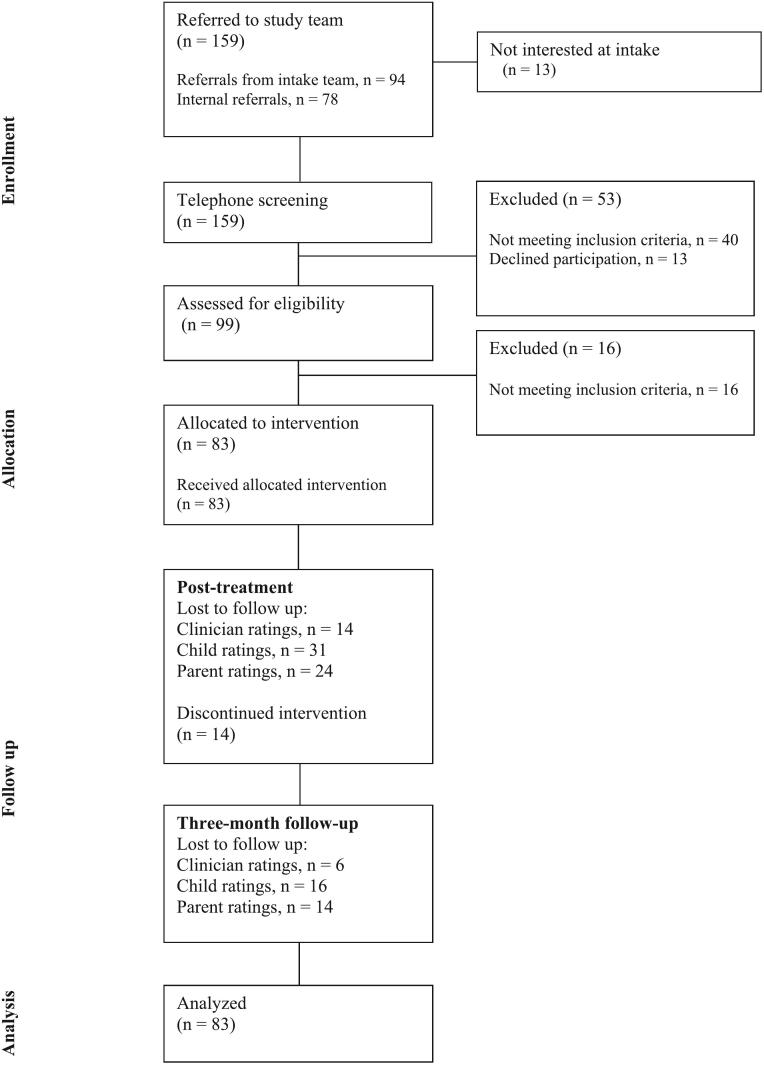
Flow diagram of the progress through the trial.

At the primary endpoint, there were 24 % missing data on the primary outcome measure, and 61 % and 46 % missing data on child- and parent reported measures, respectively. See Fig. 1 for participant flow and attrition. Missingness on the primary outcome measure was not significantly associated with baseline symptom severity (*t*(81) = −0.65, *p* = .84), age (*t*(81) = 0.13, *p* = .94) or gender (*X*^2^ (1, *N* = 83) = 2.19, *p* = .14).

### 3.2 Acceptability

#### 3.2.1 Acceptance

Fourteen (17 %) of the included participants dropped out of ICBT during the treatment period. The reasons differed from being directly related to the treatment (e.g., preferring to meet face-to-face, the program being too stressful; *n* = 3) to other circumstances (e.g., moved from the area, other problems in need of treatment; *n* = 7) or unknown (*n* = 4).

#### 3.2.2 Treatment adherence

Children had on average completed 7.5 (*SD* = 3.3) and parents 8.0 (*SD* = 3.5) out of 12 modules at post-treatment. Most children and parents, 67 (81 %) and 70 (84 %), respectively, completed at least 4 modules and thus received information about main treatment components. At post-treatment, 10 children (12 %) and 17 parents (21 %) had completed all 12 modules.

The average clinician-rated treatment adherence was moderate (*M* = 10.6, *SD* = 6.1). The average score on item 4 (how actively the participant had worked with treatment content) was 2.2 (*SD* = 1.4) at post-treatment, indicating that participants were somewhat actively working with the treatment content outside of the modules.

#### 3.2.3 Satisfaction and credibility

Of those who completed questions related to treatment satisfaction, a vast majority reported that they to some degree had learnt how to better deal with their own (87 %) or their child's (98 %) anxiety/OCD, and almost all participants (89 % of children, 97 % of parents) rated the treatment as at least “OK” (see Table 2). Ratings of treatment credibility were moderate and are presented in Supplementary Table S2.

**Table 2:** Child and parent ratings of satisfaction. Observed values and proportions.

		Child (*N* = 47) *N* (%)	Parent (*N* = 58) *N* (%)
I learnt to handle OCD/anxiety better.	Not at all	6 (12.8)	1 (1.7)
	Somewhat	17 (36.2)	32 (55.2)
	Completely	24 (51.1)	25 (43.1)
The materials were…	Too childish	6 (12.8)	3 (5.2)
	Appropriate	36 (76.6)	52 (89.7)
	Too advanced	5 (10.6)	3 (5.2)
The amount of therapist contact was…	Too seldom	2 (4.3)	6 (10.3)
	Appropriate	43 (91.5)	52 (89.7)
	Too much	2 (4.3)	0 (0)
The internet format…	Suited me	28 (59.6)	23 (39.7)
	Was good but I would have liked to meet occasionally	14 (29.8)	31 (53.4)
	I would have preferred face-to-face treatment	5 (10.6)	4 (6.9)
Overall, the internet treatment was…	Bad	5 (10.6)	3 (5.2)
	Ok	15 (31.9)	16 (27.6)
	Good	17 (36.2)	28 (48.3)
	Great	10 (21.3)	11 (19.0)

OCD=Obsessive-Compulsive Disorder.

### 3.3 Feasibility

#### 3.3.1 Therapist support

Therapist time was on average 13.03 (*SD* = 13.48) minutes per week for children and 15.2 (*SD* = 13.7) for parents. In total, 29 participants (35 %) were given additional support between baseline and the three-month follow-up. Of these, 15 participants received face-to-face sessions (*M* = 1.9, SD = 2.4, *Mdn* = 1, range 1–10) and 14 participants received phone calls (*M* = 1.2, *SD* = 0.4, *Mdn* = 1, range 1–2). The reasons for additional sessions or phone calls were either directly related to treatment (e.g., working with or planning exposure, exposure hierarchies, problem solving, or parental support, *n* = 14) or concerning other issues (e.g., suicidal thoughts, weight loss, neuropsychiatric testing, school meetings, or medication, *n* = 19).

#### 3.3.2 Adverse events

No serious adverse events were reported during the trial. One or more adverse event was reported from 16 of the 46 children (33 %) and 16 of the 57 parents (27 %) who completed the post-treatment assessment. Adverse events included e.g., feelings of stress and pressure to work with ICBT, increase in obsessive thoughts and reassurance seeking and acts of self-harm. Most children (62 %) and parents (63 %) who reported adverse events rated them as having little or no negative impact at post-treatment. However, three children (19 %) and two parents (13 %) reported that the adverse events still had a large negative impact on the participants well-being at post-treatment.

Additionally, five of 69 participants (7 %) were assessed as deteriorated as measured by therapists on the CGI-I at post-treatment. The corresponding proportion at three-month follow-up was 10 % (6/63 participants).

### 3.4 Effectiveness

#### 3.4.1 Clinical outcome measures

We observed a statistically significant effect of time (*B* [SE] = −0.92 [0.09]; *p* < .001), with a large model-implied within-group effect size (*d* = 2.45), on the primary outcome (CGI—S). As shown in Table 3, participants improved significantly from baseline to follow-up on all measures. Completer analyses are presented in the Supplementary material.

**Table 3:** Observed means and standard deviations for outcome measures at all assessment points, and estimated change between baseline and three-month follow-up, including effect sizes.

Outcome	Observed mean (SD)			Baseline-FU	
	Baseline	Post	Follow-up	Estimated change	Cohen's d [95 % CI]
CGI-S				−1.84⁎⁎⁎	2.45 [2.02; 2.88]
*m*	4.80	3.72	2.97		
*sD*	(0.75)	(0.13)	(1.53)		
*n*	83	68	63		
CGAS				9.36⁎⁎⁎	1.37 [1.01; 1.73]
*m*	54.80	63.34	63.65		
*sD*	(6.85)	(11.18)	(13.98)		
*n*	82	64	60		
CYBOCS				−11.48⁎⁎⁎	2.93 [1.88; 3.98]
*m*	23.81	16.40	13.30		
*sD*	(3.92)	(8.80)	(7.09)		
*n*	16	15	10		
RCADS-C-ANX				−10.6⁎⁎⁎	0.55 [0.19; 0.91]
*m*	40.58	36.15	29.78		
*sD*	(19.10)	(20.92)	(17.27)		
*n*	79	48	32		
RCADS-P-ANX				−9.94⁎⁎⁎	0.70 [0.36; 1.04]
*m*	38.82	32.37	28.29		
*sD*	(14.10)	(15.23)	(16.15)		
*n*	83	59	45		
EWSAS-C				−5.4⁎⁎⁎	0.66 [0.30; 1.02]
*m*	15.70	12.74	10.50		
*sD*	(8.14)	(9.70)	(10.40)		
*n*	79	47	32		
EWSAS-P				−7.52⁎⁎⁎	1.03 [0.67; 1.38]
*m*	18.00	12.85	10.37		
*sD*	(7.33)	(8.94)	(8.60)		
*n*	83	59	46		

Abbreviations. FU = Three-month follow-up; CI = Confidence interval CGI-S = Clinical Global Impression – Severity; CGAS = Children's Global Assessment Scale; CY-BOCS = Children's Yale-Brown Obsessive Compulsive Scale; RCADS-C/P-anx = Revised Children's Anxiety and Depression Scale, anxiety subscales – child and parent version; EWSAS = The Education, Work and Social Adjustment Scale – child and parent version. Note. Higher scores on CGAS indicate higher functioning.

⁎⁎⁎ *p* < .001.

#### 3.4.2 Improvement and remitters

Based on available data, twenty-eight participants (34 %) showed at least “much” improvement on the CGI-I at post-treatment. The proportion had increased to 33 participants (40 %) at three-month follow-up. Twenty-five percent of participants were classified as remitters at post-treatment and the proportion had increased to 43 % at the three-month follow-up.

#### 3.4.3 Further need of treatment

At the three-month follow-up, 31 participants (37 %) could be discharged from the clinic. Eleven participants (13 %) needed further support or treatment for the disorder that had been targeted in the ICBT program. Other reasons for needing further contact with the clinic were support concerning an anxiety disorder or OCD that was not the focus during ICBT (*n* = 4), depressive symptoms (*n* = 5), neuropsychiatric assessment (*n* = 6), other reason (e.g., eating disorder, self-harm, follow-up on medication; *n* = 11) or unknown (*n* = 15).

#### 3.4.4 Long-term follow-up

Of the 66 participants who consented to long-term follow-up, 38 (46 %) had been in contact with CAMHS during the two years after post-treatment. On average, they received 21 sessions (*SD* = 22.1; *Mdn* = 15.5). Reasons for contact were assessment or management of a neuropsychiatric disorder (*n* = 10), anxiety disorder and/or OCD (*n* = 10), depressive symptoms (*n* = 3), or other/mixed (*n* = 15).

### 3.5 Predictors of outcome

Only baseline CGAS remained significantly associated with improvement in a multiple regression (*F* (4,48) = 3.48, *p* = .014). See Supplementary Tables S3 and S4 for results.

## 4 Discussion

The aim of this study was to conduct a naturalistic evaluation of the BIP Anxiety and BIP OCD programs within a non-specialized CAMHS setting. We found ICBT to be an acceptable and feasible treatment option for children and adolescents when delivered in this context. Results suggested that ICBT could potentially be effective, but given the large amount of missing data, these results must be interpreted with great caution. Based on completer analyses, treatment improvement was highest among children with higher global functioning at baseline.

Regarding feasibility, therapist time (in total 28 min per family) was slightly higher compared to previous ICBT studies who have showed therapist times of around 20 min (e.g., Aspvall et al., 2020; Aspvall et al., 2021a, Aspvall et al., 2021b; Jolstedt et al., 2018a; Jolstedt et al., 2018b; Lenhard et al., 2017). The larger amount of therapist time could reflect a more heterogeneous study sample or perhaps the therapists' relative inexperience with ICBT. Only a few of the therapists were involved during the whole study period and thus did not have time to become skilled ICBT therapists. Difficulties managing ICBT as well as their ordinary clinical workload were one stated reason for therapist turnover. Although not a predefined measure of feasibility, this suggests that how ICBT is organized, e.g., in specialized teams, might be important for successful implementation.

Interestingly, only a small proportion of participants responded that they would have preferred face-to-face treatment, even though most participants did not live far away from the clinic. However, 18 % received additional parallel face-to-face sessions, which might have influenced the ratings.

Although all measures showed statistically significant improvements and almost half of the study sample were free of their principal diagnosis at the primary endpoint, most participants still experienced symptoms after the end of treatment, and remission rates were lower than in previous studies (Jolstedt et al., 2018b; March et al., 2009; Spence et al., 2011; Waite et al., 2019). Many patients also needed continued contact with CAMHS after ICBT. Results showed that lower global functioning at baseline was significantly associated with poorer treatment outcome at the three-month follow-up. Although mean global functioning at baseline was lower in the present study than in some previous studies on ICBT (e.g., Vigerland et al., 2016a, Vigerland et al., 2016b; Jolstedt et al., 2018b), it was slightly higher than in e.g., March et al. (2009), Spence et al. (2011) and Waite et al. (2019). This suggests that sample severity does not explain the relatively low remission rates. Therapist experience and comorbidity could be other important factors. Indeed, the long-term follow-up, showing that 46 % of children had continued contact with CAMHS mainly for reasons other than those targeted in ICBT, indicates that the needs of the sample were greater than the scope of ICBT.

Despite this, there were a large proportion of patients for whom ICBT was effective, even if improvements did not always lead to remission. These results raise the question of how ICBT should best be organized within routine clinical care. One way of implementing ICBT is in a stepped-care model. This approach has been shown to be an effective and cost-effective way of delivering treatment both for anxiety disorders (Rapee et al., 2017; Chatterton et al., 2019) and OCD (Aspvall et al., 2021a; Aspvall et al., 2021b) and should be further evaluated in non-specialist CAMHS.

Although no serious adverse events were reported during the trial, three children (19 %) and two parents (13 %) reported that their adverse events still affected them negatively at post-treatment. This highlights the importance of monitoring progress in ICBT and discontinuing the intervention if needed.

Strengths of this trial were a consecutive inclusion over a two-year period in a non-specialized CAMHS setting, a two-year follow-up, the use of several informants and well-validated measures. We included participants with a range of comorbid disorders, who had not sought out ICBT specifically, and clinicians with minimal training in ICBT provided therapist support. This strengthens the external validity, and the results can probably be generalized to patients and therapists in other regular CAMHS.

This study also has several limitations. Most importantly, the lack of a control group means we cannot rule out that participants improved due to passage of time. Indeed, Waite et al. (2019) found no significant difference between ICBT and a waitlist control for adolescent anxiety disorders in a regular CAMHS. Furthermore, assessments were not conducted by masked clinicians, and clinicians received minimal training in assessment procedures and had limited experience of using the clinician rated measures outside of the trial. Remission was based on a one-item clinician rated measure, and not on child- and parent-ratings, and several measures were not well-validated but chosen for practical reasons. Another important limitation in the study is the amount of missing data, especially on child- and parent-ratings at primary endpoint. Mixed-models, like all traditional statistical approaches, yield biased estimates if data are not missing at random (Gueorguieva and Krystal, 2004). Given the large amount of missing data, the results on effectiveness must be interpreted with caution. Supplementary completer analyses still show statistically significant improvements but smaller effect sizes for clinician rated outcomes. The predictors analyses were conducted on a relatively small sample and without handling missing data and must therefore also be interpreted with caution. Furthermore, organizational barriers to implementation were not formally assessed, partially hampering our ambition to evaluate ICBT as it might be used if it were broadly disseminated.

In summary, the evaluation of ICBT in non-specialist CAMHS found ICBT to be acceptable, feasible and potentially effective. However, given the limitations of the naturalistic study design, further studies are needed to confirm treatment effectiveness in this setting. Further research is also required to better understand if ICBT is a cost-effective method in non-specialist CAMHS, and if so, how to organize it most efficiently.

## Funding

This study was funded by The 10.13039/501100006636Swedish Research Council for Health, Working Life and Welfare (Forte 2014-4052). The funder was not involved in planning the study design, collecting data or interpreting results. The study was also supported by 10.13039/501100009778Region Jämtland Härjedalen, who was not involved in interpreting and reporting results.

## Declaration of competing interest

The authors declare the following financial interests/personal relationships which may be considered as potential competing interests: Mataix-Cols receives royalties for contributing articles to UpToDate, Inc., outside the submitted work. Other authors report no conflicts of interest.

## Appendix A Supplementary data

Supplementary material Image 1
