# Supplementary material for: Effectiveness of internet-delivered cognitive behavioural therapy for anxiety and obsessive-compulsive disorders within routine clinical care in rural Sweden
Source: Internet Interv. 2024 Apr 6;36:100738. doi: 10.1016/j.invent.2024.100738 (PMC11015440; doi:10.1016/j.invent.2024.100738)
Supplement: Supplementary file 1 — Supplementary material [file mmc1.docx]

**Supplementary material**

Supplement 1. Detailed description of measures.

**Measures of acceptability**

Treatment adherence was measured by the number of completed modules and the clinician rated questionnaire Internet Intervention Patient Adherence Scale (iiPAS; Lenhard et al., 2019). The iiPAS consists of five items regarding 1) tempo, 2) engagement in exercises, 3) communication with the therapist, 4) motivation to test strategies and 5) frequency of logins which is rated on a 5-point scale from 0-4, yielding a maximum total score of 20 points. Higher scores correspond to higher adherence. Ratings were completed at mid-treatment and post-treatment. Since these ratings were highly correlated, *r*(48) = .77, *p <* .01, only the post-treatment measurement was used for subsequent analyses.

Treatment credibility was measured three weeks after starting the program, using the Treatment credibility and expectancy – child and parent version (C-scale), which consists of five items regarding treatment credibility (if they found the treatment logical, if they thought it would be successful in treating their difficulties, if they would recommend the treatment to a friend with similar problems, how effective it would be for another fear or worry and how much they expected to improve), which are rated on a scale from 0 = ”Not at all sure” to 10 = “Completely sure” (Borkovec & Nau, 1972).

**Measures of effectiveness**

Improvement was rated using the Clinical Global Impression - Improvement (CGI-I; Guy, 1976). The measure is rated on a seven-point scale from 1 = “Very much improved” to 7 (¨Very much worse”).

The Children's Global Assessment Scale (CGAS; Shaffer, 1983) was used by the clinician to rate global functioning on a scale from 0 to 100, with higher scores corresponding to higher functioning.  It has shown moderate to excellent inter-rater reliability and good validity (Bird et al., 1987; Lundh et al., 2010).

Symptoms of depression and anxiety were measured with the Revised Children's Anxiety and Depression Scale - Child and parent version (RCADS-C/P; Chorpita et al., 2005), which consists of 47 items rated on a four-point scale from 0 (“Never”) to 3 (“Always”), and has been found to show acceptable convergent, discriminant, and factorial validity. Only the anxiety subscale (31 items) is used for the analyses of effectiveness in this study. Higher scores indicate more anxiety.

When OCD was the disorder targeted in treatment, the CY-BOCS (Scahill et al., 1997) was administered to assess OCD symptom severity. It consists of a checklist and a 10-item severity rating, yielding a total score of 0-40, with higher scores corresponding to higher symptom severity. It has shown reasonable reliability and validity and good to excellent inter-rater agreement (Scahill et al., 1997).

The Work and Social Adjustment Scale – youth and parent version (WSAS-Y/P; Jassi et al., 2019) was used to assess impairment. It consists of five items which are rated on a 9-point scale from 0-8. Higher scores indicate more impairment. The scale has shown adequate test-retest reliability and good convergent/divergent validity.

Need of further treatment was assessed by the therapist at the three-month follow-up. The decision was made in line with the clinicians' usual clinical procedures at the clinic and based on a general assessment of overall severity, functioning, and patient motivation.

Supplementary Table S1.

*Overview of treatment content*

|  | **BIP Anxiety** | | | **BIP OCD** | |
| --- | --- | --- | --- | --- | --- |
| Module | Child/Adolescent | Parent (child version) | Parent (adolescent version) | Child/Adolescent | Parent |
| 1 | Introduction to ICBT. Psychoeducation about fear and anxiety, safety behaviours and avoidance. Introducing functional analysis. | Introduction to ICBT. Psychoeducation about fear and anxiety, safety behaviours and avoidance. Introducing functional analysis. | Introduction to ICBT. Psychoeducation about fear and anxiety, safety behaviours and avoidance. Introducing functional analysis. | Introduction to ICBT. Psychoeducation about OCD. Functional analysis. | Introduction to ICBT. Psychoeducation about OCD. Functional analysis. |
| 2 | Coping. Continued functional analysis. | Coping. Preparing for goal setting and exposure hierarchies. | Parental behaviours that maintain anxiety. | Introduction to CBT, exposure and goal setting. | Introduction to CBT, exposure and goal setting. |
| 3 | Goal setting and exposure hierarchies. | Preparing for exposure, rewards, managing possible obstacles. | Preparing for exposure, rewards, managing possible obstacles. | Exposure and exposure hierarchies. | Exposure. |
| 4 | Preparing for exposure. | Planning for exposure. | Helpful parent strategies | Preparing for exposure. | Common reactions in parents. Functional analysis on parental behaviours. Parent strategies. |
| 5 | Plan and follow-up of exposure exercises.  Introducing interoceptive exposure | Plan and follow-up of exposure exercises. | Problem solving and managing worry. | Plan and follow-up of exposure exercises. | Plan and follow-up of exposure exercises. |
| 6 | Plan and follow-up of exposure exercises. How to manage anxious thoughts. | Plan and follow-up of exposure exercises. | Plan and follow-up of exposure exercises. | Plan and follow-up of exposure exercises. Family accommodation. | Plan and follow-up of exposure exercises. Introducing family accommodation. |
| 7-11 | Planning and follow-up of exposure exercises. Repetition of previous content. | Planning and following-up of exposure exercises. Overcoming obstacles and maintaining motivation. Chapter 8: Problem solving. | Planning and following-up of exposure exercises. Overcoming obstacles and maintaining motivation.  Chapter 7: Working with anxious thoughts. | Planning new exposure and following-up on a weekly basis.  Chapter 8: Working with obsessive thoughts. | Planning new exposure and following-up on a weekly basis. Reducing family accommodation. Overcoming obstacles and maintaining motivation.  Chapter 8: Working with obsessive thoughts. |
| 12 | Summary and evaluation of the program. Maintaining progress and continued exposure. | | | | |

*Note. Recommended pace for module completion was one per week.*

Supplementary Table S2.

| *Child and parent ratings of treatment credibility. Observed means and standard deviations from N=76 children and N=81 parents.* | | |
| --- | --- | --- |
|  | Child  *M (SD)* | Parent  *M (SD)* |
| How logical does the treatment seem? | 6.29 (2.48) | 7.75 (1.93) |
| How sure are you that this method will be successful in helping with your anxiety/OCD? | 5.22 (2.48) | 6.11 (2.03) |
| How sure are you that you would recommend this kind of treatment to a friend with similar problems? | 5.95 (2.89) | 7.0 (1.94) |
| How effective do you think this kind of treatment would be for another fear or worry? | 6.51 (2.34) | 7.26 (1.83) |
| How much do you think you will improve from this treatment? | 6.32 (2.65) | 7.09 (1.98) |

Supplementary Table S3.

*Correlation between improvement at three-month follow-up and demographic, baseline and in-treatment variables.*

| Variables | Improvement (CGI-I) |
| --- | --- |
| *Demographic variables*  Age  Duration of disorder  *Baseline variables*  CGI-S  CGAS  RCADS-C_tot  RCADS-C _anx  WSAS-Y  RCADS-P_tot  RCADS-P_anx  WSAS-P  *In treatment variables*  Therapist time_C  Therapist time_P  iiPAS_tot  iiPAS item 4  Completed chapters-C  Completed chapters-P | .22  .19  -.004  -.311*  .003  -.001  .12  .02  .000  .14  .08  -.05  -.31*  -.26  -.34*  -.34* |

*Abbreviations*. CGI-S = Clinical Global Impression – Severity; CGAS = Children’s Global Assessment Scale; CY-BOCS = Children’s Yale-Brown Obsessive Compulsive Scale; RCADS-C/P-anx = Revised Children’s Anxiety and Depression Scale, anxiety subscales – child and parent version; WSAS = The Work and Social Adjustment Scale – youth and parent version. *Note*. Higher scores on CGAS (Children’s Global Assessment Scale) indicate higher functioning. *** p < 0.001.

Supplementary Table S4.

*Regression mode fit and coefficients for variables significantly correlated with improvement.*

| Variables | Improvement (CGI-I) | |
| --- | --- | --- |
|  | *B* | *SE* |
| *Baseline variables*  CGAS  *In treatment variables*  iiPAS  Completed chapters-C  Completed chapters-P  Model fit  (R^2^_adj_) | -.05*  -8.6E-5  -.13  -.04 | .02  .05  .13  .12  *F*(4,48) = 3.48, *p*=.014  (16%) |

*Abbreviations*. CGAS = Children’s Global Assessment Scale; iiPAS=internet intervention patient adherence scale; C=Child; P= Parent. *Note*. Higher scores on CGAS (Children’s Global Assessment Scale) indicate higher functioning. *p < .05

Supplementary Table S5.
*Observed means and standard deviations for outcome measures at all assessment points, and paired sample t-test of change between baseline and three-month follow-up, including effect sizes, for completers*

| Outcome | Observed means (*SD*) | | |  | Change |  |  |
| --- | --- | --- | --- | --- | --- | --- | --- |
|  | *Baseline* | *Post* | *Follow-up* |  | *t* | *p* | *Cohen’s d*  *[95% CI]* |
| CGI-S  *M*  *SD*  *N* | 4.80  (0.75)  83 | 3.72  (0.13)  68 | 2.97  (1.53)  63 | Pre-post  Pre-FU | 6.30  9.07 | <.001  <.001 | 0.99 [0.61;1.37]  1.49 [1.01;1.98] |
| CGAS  *M*  *SD*  *N* | 54.80  (6.85)  82 | 63.34  (11.18)  64 | 63.65  (13.98)  60 | Pre-post  Pre-FU | -6.55  -5.64 | <.001  <.001 | -.89 [-1.20;-0.57]  -.75 [-0.43;-1.06] |
| CYBOCS  *M*  *SD*  *N* | 23.81  (3.92)  16 | 16.40  (8.80)  15 | 13.30  (7.09)  10 | Pre-post  Pre-FU | -3.14  -3.30 | .005  .008 | 1.01 [0.25;1.77]  1.81 [0.31;3.3] |
| RCADS-C-ANX  *M*  *SD*  *N* | 40.58  (19.10)  79 | 36.15  (20.92)  48 | 29.78  (17.27)  32 | Pre-post  Pre-FU | 2.62  4.12 | .006  <.001 | 0.22 [0.06;0.37]  0.59 [0.28;0.90] |
| RCADS-P-ANX  *M*  *SD*  *N* | 38.82  (14.10)  83 | 32.37  (15.23)  59 | 28.29  (16.15)  45 | Pre-post  Pre -FU | 4.76  4.64 | <.001  <.001 | 0.44 [0.22;0.65]  0.69 [0.39;1.0] |
| EWSAS-C  *M*  *SD*  *N* | 15.70  (8.14)  79 | 12.74  (9.70)  47 | 10.50  (10.40)  32 | Pre-post  Pre -FU | 3.11  3.79 | .002  <.001 | 0.32 [0.10;0.23]  0.54 [0.23;0.86] |
| EWSAS-P  *M*  *SD*  *N* | 18.00  (7.33)  83 | 12.85  (8.94)  59 | 10.37  (8.60)  46 | Pre-post  Pre -FU | 6.35  6.61 | <.001  <.001 | 0.62 [0.38;0.85]  0.95 [0.61;1.28] |

*Abbreviations*. FU= Three-month follow-up; CI = Confidence interval; CGI-S = Clinical Global Impression – Severity; CGAS = Children’s Global Assessment Scale; CY-BOCS = Children’s Yale-Brown Obsessive Compulsive Scale; RCADS-C/P-anx = Revised Children’s Anxiety and Depression Scale, anxiety subscales – child and parent version; EWSAS = The Education, Work and Social Adjustment Scale – child and parent version. *Note*. Higher scores on CGAS indicate higher functioning. One sided *t*-test. *** p < 0.001.
